# Temporal Learning and List-Level Proportion Congruency: Conflict Adaptation or Learning *When* to Respond?

**DOI:** 10.1371/journal.pone.0082320

**Published:** 2013-11-28

**Authors:** James R. Schmidt

**Affiliations:** Department of Experimental Clinical and Health Psychology, Ghent University, Ghent, Belgium; University of Groningen, The Netherlands

## Abstract

The current report presents a temporal learning account as a potential alternative to the conflict adaptation account of list-level proportion congruent effects in the Stroop paradigm. Specifically, retrieval of information about response times on previous trials influences a participant's preparedness to respond at a similar time on following trials. First, an adaptation of the Parallel Episodic Processing (PEP) model is presented, and a list-level effect is produced with a temporal learning mechanism. Next, linear mixed effect model analyses show that temporal learning biases are present in list-level proportion congruent data. A non-conflict experiment is then presented in which a list-level effect is observed with a contrast, rather than congruency, manipulation. Analyses of the experimental and simulated data could not, however, provide a clear picture of whether temporal learning was the sole contributor to the list-level proportion congruent effect. These results do, however, demonstrate that caution is warranted when interpreting list-level proportion congruent effects.

## Introduction

The ability to learn the relations (or contingencies) between events in our environment is crucial for interacting with the world [1]. Perhaps equally fundamental is the knowledge of how things covary in time. Knowing the series of notes in a song, for instance, is insufficient information to play it if we know nothing about the timing and duration of said notes. Like the concept of space-time in physics, all human actions are actions occurring in time. The *when* is just as important as the *what.* It has already been argued that participants encode not only what stimuli we present them, but also temporal information about these stimuli [2]. Furthermore, knowledge about when to respond has important influence over performance in speeded response time tasks [3]–[6]. In the current work, it is argued that temporal learning might be able to explain an experimental finding that was previously interpreted as strong evidence for conflict adaptation: the list-level proportion congruent effect.

### Standard, Item, and List Proportion Congruency

In the Stroop paradigm, participants must ignore the identity of a distracting word and respond to the colour it is presented in [7]. Response times are slower when the word and colour are incongruent (e.g., the word RED presented in blue; RED_blue_) relative to when they are congruent (e.g., RED_red_). The size of this congruency effect is further influenced by the proportion of congruent trials in the task. Specifically, the effect is much larger if most of the trials are congruent (e.g., 70% congruent, 30% incongruent), rather than incongruent (e.g., 30% congruent, 70% incongruent). This proportion congruent (PC) effect is typically interpreted in terms of *conflict adaptation* [8]–[11]. Specifically, it is argued that when most of the trials are incongruent participants attempt to avoid further conflict by minimizing attention to the source of conflict (viz., the distracting word), thus resulting in a smaller congruency effect. In contrast, when there are very few conflict trials, attention to the word is allowed, resulting in a larger congruency effect.

In recent years, however, some concerns with the conflict adaptation account have been raised. For instance, Jacoby, Lindsay, and Hessels introduced the item-specific PC task [12]. Instead of manipulating PC between-participants or between-blocks, it was manipulated between items. That is, some words (e.g., BLUE and RED) were presented most often in their congruent colour, while others (e.g., GREEN and YELLOW) were presented most often in an incongruent colour. A larger congruency effect was observed for mostly congruent items. The conflict adaptation account says that PC effects are due to modulation of attention to the word in response to conflict, but given that high and low PC trials are intermixed in the item-specific preparation a participant cannot know at the start of a trial whether they need to attend or not attend to the word. Said differently, participants do not know whether the word is mostly congruent or incongruent until they have already identified it (and therefore attended to it).

Schmidt and colleagues [13]–[16] have argued that item-specific PC effects are due to contingency learning. Specifically, mostly congruent words predict (and therefore facilitate) the congruent response (e.g., BLUE is presented most often in blue), leading to a larger congruency effect. Mostly incongruent words predict (and therefore facilitate) an incongruent response (e.g., YELLOW is presented most often in orange), leading to a smaller congruency effect. Thus, contingencies between words and responses can explain the item-specific PC effect.

While debate still continues as to whether contingencies are the whole story in the item-specific PC task, a separate issue is whether PC effects can be observed at the list-level. A list-level PC effect is a proportion congruent effect that is driven by the PC of the task as a whole, rather than by specific items. Recent work has shown that, while the majority of the PC effect is explained by item-specific learning, list-level PC effects can also be observed [17], [18]. For instance, Hutchison found that *critical items* that do not vary in PC across conditions that are presented along with other congruent *filler items* (list-level mostly congruent) will have a larger congruency effect than identical critical items presented along with incongruent filler items (list-level mostly incongruent). Thus, the PC effects for these critical items cannot be due to any sort of item-specific learning (e.g., contingency learning), as it is the filler items that set the PC level. This effect for the contingency-unbiased critical items, the list-level PC effect, is easily explainable by the conflict adaptation account, making it a critical finding in the debate about whether or not conflict adaptation is observable.

It is important to note the differences between the standard, item-specific, and list-level PC effects. The standard PC task confounds item-specific and list-level PC, because *all* items are presented most often in their congruent colour in the mostly congruent condition, whereas *all* items are presented most often in their incongruent colour in the mostly incongruent condition. Thus, the standard PC paradigm is ambiguous, not allowing us to know whether an observed effect is item- or list-based. The item-specific PC task removes all list-level biases and focuses specifically on item-specific biases. Neither of these two effects are of interest in the current report. Instead, this report focuses on the list-level PC task, which removes all item-specific biases and looks for a PC effect within contingency-unbiased items.

### The Temporal Learning Hypothesis

The list-level PC effect may seem to provide powerful evidence for task-wide conflict adaptation. However, there may be yet another simple learning bias that could account for the effect. The proposal of the current work is that list-level PC effects might be explained by participants learning *when* to respond (i.e., temporal learning), rather than learning *what* to respond (i.e., contingency learning). Learning about when to respond is indeed fundamental to all human behaviour. Whether learning when to release a baseball in a throwing motion, the timing of notes in a song, or, more incidentally, when to anticipate a key press response in a psychological experiment, time is an integral part of all learning.

How does this learning occur? According to the *temporal coding hypothesis* we store in our memory of past events not only information about stimuli and responses, but also information about the timing of events [2]. Of particular importance, information about the latency between stimulus onset and when a participant responds (i.e., response time) might be encoded. Further work shows that temporal information is used by participants in an anticipatory way on following trials. For the purposes of the current paper, this is referred to as the *temporal learning hypothesis*. For instance, the literature on mixing costs provides good evidence that speed of responding on previous trials strongly influences the speed of responding on subsequent trials [19]. Performance in *pure lists*, where there is one block of all easy items and another block of all hard items, is compared with performance in *mixed lists*, where there is a single block containing both easy and hard items intermixed. The difficulty effect (i.e., the difference in performance between easy and hard items) is reduced in mixed lists relative to pure lists. There are various explanations for such mixing costs [20]–[25], but what seems clear is that fast responses to easy items affect slow responses to hard items, and vice versa. The same should be true of fast and slow responses to, respectively, congruent and incongruent trials.

**Figure 1:**
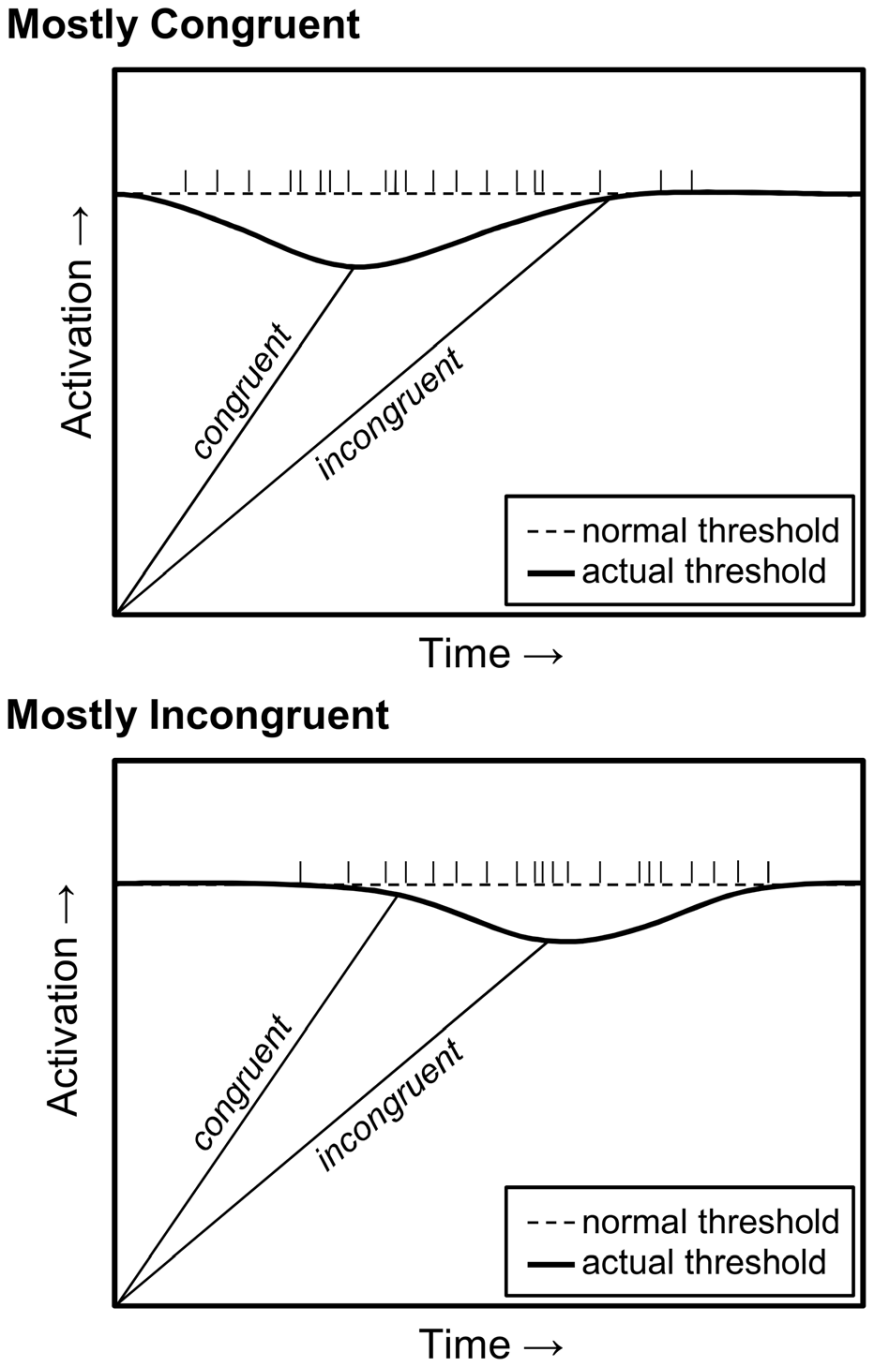
An illustration of temporal learning via anticipatory drops in the response threshold. The threshold drops earlier in the mostly congruent condition (top panel), benefiting congruent trials. The threshold drops later in the mostly incongruent condition (bottom panel), benefiting incongruent trials. Vertical tick marks on the normal threshold represent retrieved response times.

The novel suggestion of the current report is that the list-level PC effect may be produced by participants retrieving stored information about when to respond and using this information to prepare for the moment when they are ready to output a response. For instance, if a memory search reveals that most of the previous trials were responded to quite quickly, then participants will be most prepared to respond during that same (fast) response window [26]. As illustrated in Figure 1, this preparedness leads to a decrease in the response threshold at time periods that closely match a number previous response times. This will mean that it will be easier to output a response at a similar time as previous trials, resulting in rhythmic responding [27].

In the mostly congruent condition (top panel of Figure 1), this rhythm will be fast and will benefit congruent responses. The response will be ready at the expected time, enabling a fast response. On infrequent incongruent trials, response activation will be too weak to cross the temporarily reduced threshold, resulting in no advantage. A large congruency effect will therefore be observed. In the mostly incongruent condition (bottom panel of Figure 1), most previous responses are slow, leading to a slower expectancy. Congruent trials still take less time than incongruent trials due to the lack of conflict, but participants are less prepared for a quick response due to the slower rhythm they are in. Incongruent trials benefit from the slower expectancy, however, because the response is available at the expected time (i.e., when the response threshold is lowered). This results in a small congruency effect.

In sum, the faster rhythm in the mostly congruent condition will lead to a larger congruency effect than the slower rhythm in the mostly incongruent condition. An interaction between PC and congruency is therefore produced simply because participants have learned different expectations about *when* to respond in the two PC conditions. Note that this temporal expectancy will be at the list- rather than item-level, because episodic retrieval decreases the global response threshold for all responses. It is also important to realize that this mechanism will produce a similar pattern of results in errors, as the decrease in the response threshold in the mostly congruent condition will increase the propensity for fast incongruent errors. Thus, a list-level PC effect in errors is expected, as observed by Hutchison [18]. The first goal of this paper is to demonstrate computationally that temporal learning can produce a list-level PC effect.

## Analysis 1: Simulated List-Level PC

**Figure 2:**
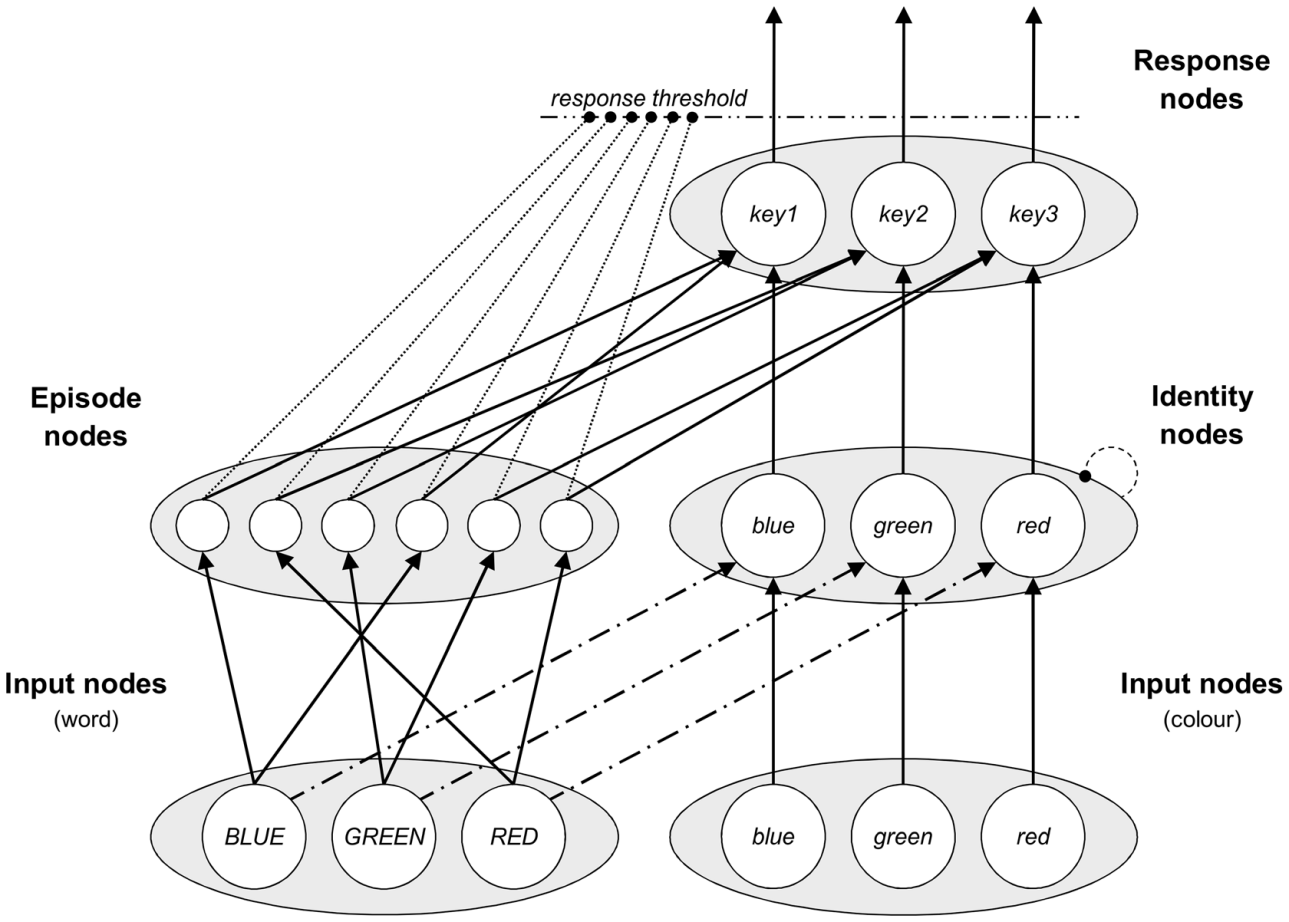
The structure of the Parallel Episodic Processing (PEP) model. Input nodes are stimulated first. Words and colours compete in Identity nodes, before passing activation on to Response nodes. Words also activate Episode nodes, which then activate the associated Response nodes. New to the model, Episode nodes also affect the response deadline dynamically over the course of a trial.

The Parallel Episodic Processing (PEP) model [14] was adapted to learn information about time. A representation of the model is presented in Figure 2. In this model, colour and word Input nodes feed activation into Identity nodes, where conflict occurs, and then on to Response nodes. Word Input nodes also feed activation into Episode nodes. On each trial, a new Episode node is made, which links together the stimuli presented with the response that was made. Thus, a given word Input node will activate the Episode nodes that it is linked to (i.e., from trials in which that word was presented), and these Episode nodes will then bias the Response nodes that they are connected to. These simple storage and retrieval processes therefore produce contingency learning. In order to allow the model to learn about time, the model was adjusted to record the response time of the model into each episode. On subsequent trials, the response times of previously-experienced episodes are retrieved and collectively bias the global response threshold for the Response nodes. The exact changes to the model are explained in Appendix S1, but the most important detail to understand is that the response deadline is decreased the most during moments at which a large percentage of recent responses were made, with the most recent episodes having the largest influence, similar to a recent adaptation of the ACT-R framework [5].

The model is then used to simulate the list-level PC effect observed by Hutchison [18]. The expectation is for an earlier dip in the response threshold in the mostly congruent condition that benefits congruent trials, thereby increasing the congruency effect. In the mostly incongruent condition, a later dip will benefit incongruent trials instead, thereby decreasing the congruency effect. It is important to note that the model has no means to monitor or adapt to conflict. The temporal and contingency learning mechanisms are blind to congruency and PC. Furthermore, there is no attentional modulation system in the PEP model. Thus, conflict adaptation is a priori impossible. Any observation of a list-level PC effect is thus necessarily driven by temporal learning.

### Method

Fully documented source code for this (and the previous) version of the PEP model is available on the author's webpage (http://users.ugent.be/~jaschmid/PEP/). The Appendix in Schmidt [14] explains the precise math of the original model in detail, and Appendix S1 in the current report describes the changes made to the model.

#### Materials and design

The PEP model was presented with the exact same manipulations as those used by Hutchison [18], save that only one of the two mostly incongruent list types was used (viz., “Filler_single_” in Hutchison's notation). A total of 2000 simulated “participants” were run, half in the mostly congruent condition and half in the mostly incongruent condition. The two filler colour words were presented 30 times each in their congruent colour in the mostly congruent condition. These same filler words were presented 30 times in the opposite incongruent colour in the mostly incongruent condition. Note that filler items are differently biased between mostly congruent and mostly incongruent PC participants, and are thus not analysed. The remaining four critical colour words had equivalent cell frequencies in both conditions. These critical items are the items of interest in assessing a list-level PC effect. The exact cell frequencies are presented in Table 1. Note that in this procedure of Hutchison the critical items do vary in item-specific contingencies, but these cell frequencies are the same in the mostly congruent and mostly incongruent conditions. Thus, only filler items (which are not analysed) vary between the two groups of participants. Like the actual experiment, each simulated participant received 180 trials in a different randomized order.

**Table 1:** Frequencies of critical items, and mostly congruent and mostly incongruent filler items in the list-level PC manipulation.

	Critical Items				Mostly		Mostly Incongruent			
	(within manipulation)				Congruent		Single		Mixed	
Colour	yellow	blue	red	black	green	white	green	white	green	white
yellow	20	2	1	1					6	6
blue	2	20	1	1					6	6
red	2	2	10	16					6	6
black	2	2	16	10					6	6
green	2	2	1	1	30			30		6
white	2	2	1	1		30	30		6	

### Results

Given the large number of simulations per condition, reliability was high enough that statistics are not reported. Note, however, that any of the numerical differences interpreted here were well below the conventional alpha level.

#### Cycle times

**Figure 3:**
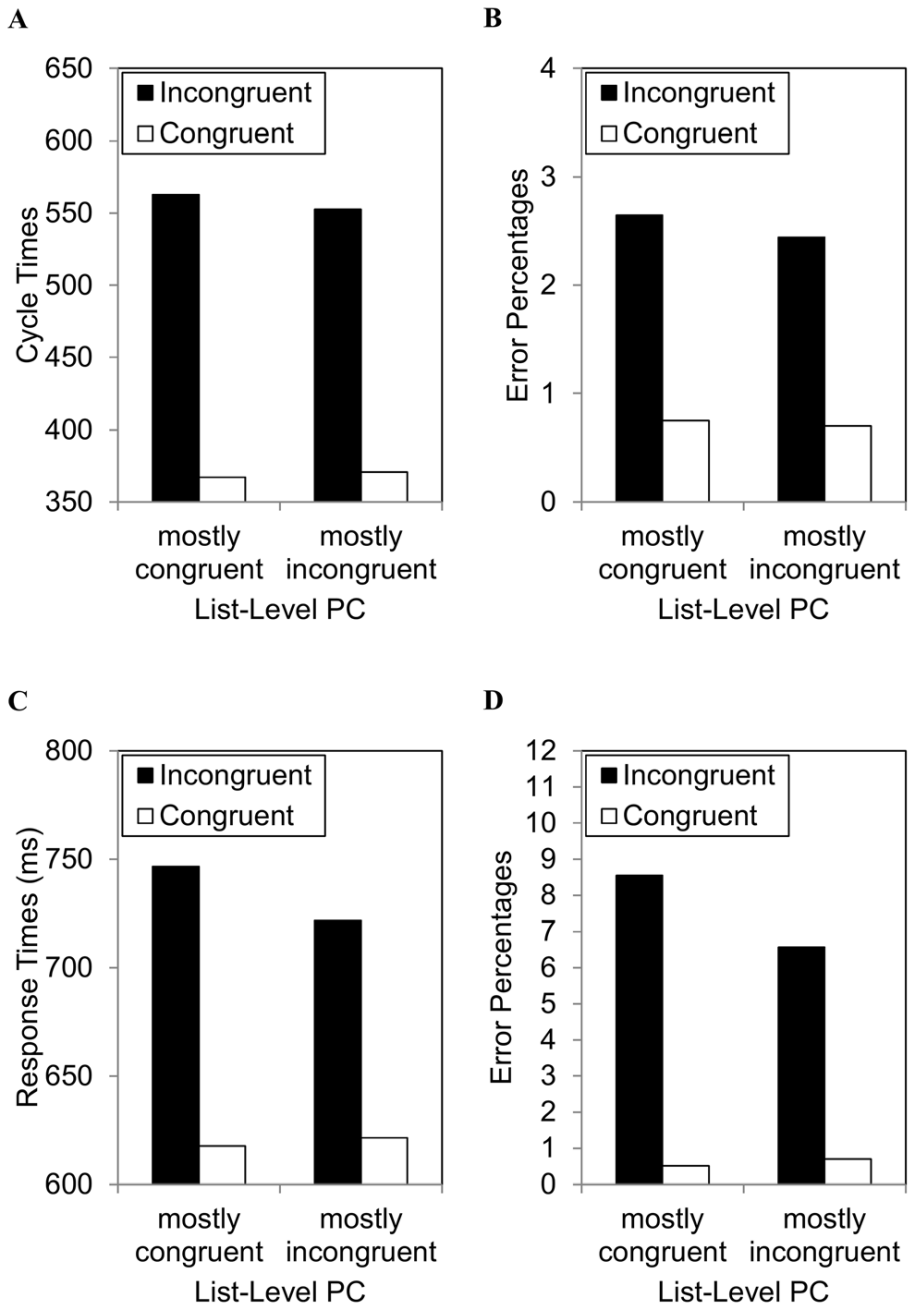
Analysis 1 data for congruency and proportion congruency. Model-simulated (A^rpar; cycle times and (B) error percentages. For comparison, the original experimental (C) response times and (D) error percentages adapted from Hutchison (2011).

The correct cycle times (i.e., simulated response times) are presented in Figure 3a. For comparison, participant response times are presented in Figure 3c. The model produced congruency effects of 196 cycles in the mostly congruent condition (congruent: 367; incongruent: 563) and 184 in the mostly incongruent condition (congruent: 371; incongruent: 553). Thus, a 14 cycle list-level PC effect was observed. Like the participant data, this was primarily driven by the incongruent trials.

#### Percentage error

The error percentages are presented in Figure 3b. For comparison, participant errors are presented in Figure 3d. Errors were relatively infrequent in the model, but consistent with the cycle times. The model produced congruency effects of 1.90% in the mostly congruent condition (congruent: .75%; incongruent: 2.65%) and 1.74% in the mostly incongruent condition (congruent: .70%; incongruent: 2.44%). Thus, a .16% list-level PC effect was observed.

### Discussion

Analysis 1 demonstrated computationally that temporal learning can produce an apparent list-level PC effect. Most critically, conflict adaptation processes are impossible in this episodic learning model. The exact numerical differences reported here were not large. This primarily has to do with the fact that parameters were not played with to perfectly match the pattern of the data. More important is the principle demonstrated here, namely, that temporal learning processes will produce an apparent list-level PC effect incidentally. It should also be noted that most of the effect was in the incongruent condition, whereas the description of Figure 1 in the Introduction might have suggested an effect for both congruent and incongruent trials. Figure 1 was a bit oversimplified for the purpose of illustrating how temporal learning can produce a list-level PC effect. The reason for a larger effect for incongruent relative to congruent trials in both the modelling and participant data probably has to do with the fact that temporal learning has more time to affect processing on incongruent trials.

## Analysis 2: Participant List-Level PC

At least in principle, the preceding computational modelling results demonstrate that temporal learning *could* produce a list-level PC effect with no need for conflict adaptation. Another important question, however, is whether evidence for temporal learning can be found in participant data. The focus of the current work is on list-level PC, but a contribution of temporal learning to the standard PC effect has already been observed. In the context of masked priming, Kinoshita, Mozer, and Forster analysed PC data in a linear mixed effects model in order to assess whether the response time on the previous trial had an impact on the size of the congruency effect on the current trial [28]. The adaptation to the statistics of the environment (ASE) model [29], [30], which inspired their work, is conceptually similar to the temporal learning account discussed in the current manuscript. The ASE estimates the probability of an accurate response at a given moment, basing this decision partly on information from previous trials, similar to the decision model [31]. On average, this leads to a lower threshold in easy blocks (e.g., mostly congruent) relative to hard blocks (e.g., mostly incongruent). Easy items are more affected by threshold changes than hard items [32], [33], resulting in a smaller congruency effect in the mostly incongruent, relative to mostly congruent, condition.

Most critically, both the PEP and ASE models predict that the congruency effect will be smaller the longer the reaction time was on the previous trial. This is exactly what Kinoshita and colleagues observed [28]. Of course, this was done in the context of a standard (i.e., contingency-biased) PC experiment, which does not allow us to distinguish between item-specific and list-level effects. Their experiment also used masked priming, and only found an effect for subliminal primes. The novel contribution of the current work is to investigate whether such temporal learning biases contribute to list-level PC effects, and with supraliminal, integrated stimuli.

To test for a role of temporal learning in the list-level PC task, Analysis 2 assessed the Stroop data of Hutchison [18] with a similar linear mixed effects model approach as that of Kinoshita and colleagues [28]. A critical difference from this past work is that the current analysis assessed contingency-unbiased data. The temporal learning hypothesis predicts that previous RT will not only correlate with current RT, but will also interact with congruency. Specifically, the congruency effect should be larger following faster responses than following slower responses. In other words, the Stroop effect gets larger the faster the temporal expectancy. Controlling for this interaction should lead to a reduction of the list-level PC effect. As discussed in greater detail later in the paper, it will probably not lead to an elimination of the PC effect, however, as previous RT is probably only a very rough estimate of a participant's temporal expectancy.

### Method

The linear mixed effects model was nearly identical to that of Kinoshita and colleagues [28]. Identical to that report, response times and previous response times were normalized with an inverse transformation (−1000/RT). This is required in order to prevent violations of distributional assumptions made by parametric regression. The negative numerator was used simply so that lower values corresponded to faster RTs and larger numbers to slower RTs. Also consistent with the past report, trials with response times shorter than 300 ms on the current or previous trials were deleted. This was determined via inspection of the Q-Q plots and further corrects the response time distribution. Again identical to the previous report, trials on which participants made an error on the current or previous trial were excluded from the analysis. As the goal was to study contingency-unbiased list-level PC effects, filler trials were removed from the analysis and only critical trials were assessed. All trials in which the colour or word on the previous trial matched the colour or word of the current trial were excluded to eliminate feature repetition biases [34]–[36].

Congruency and PC were coded as binary variables, with congruent and mostly congruent coded as 0, and incongruent and mostly incongruent coded as 1. Previous RT was centered on the grand mean to avoid correlation with the intercept. The mixed model included congruency (congruent vs. incongruent), PC (mostly congruent vs. mostly incongruent), previous RT, and their interactions as fixed factors. Subjects and items (the unique colour-word combinations) were included as random factors with the default variance components error structure. Analyses were run with the MIXED procedure in SPSS using maximum likelihood estimation. A total of 230 participants were used for the analysis. Participants were not excluded on the same basis as the original report (e.g., because the analysis here did not require that participants had working memory span data).

The most critical analyses are the congruency by PC interaction (PC effect) and the congruency by previous RT interaction (temporal learning effect). By including both in one model, they will control for each other. Thus, one possible result is a reduction of the PC effect as a result of including the congruency by previous RT interaction in the model. The analyses also consider the unique hypothesis that the effects of previous RT might actually be due to a confounding with previous congruency. That is, if the previous trial was congruent, then it would also (likely) be faster than if it was incongruent. Any effect of previous RT could therefore simply be a previous congruency effect in disguise.

### Results

#### Previous RT

The final model is presented in Table 2, and includes the main effects of congruency, PC, and previous RT, in addition to the interactions of PC and previous RT with congruency. Note that the parameter estimates are based on inverse response times, which are not easily converted back to regular response times. Excluding the theoretically less interesting interaction between PC and previous RT and the three-way interaction (also hypothesis irrelevant) does not significantly reduce the variance explained, *χ^2^*(2) = 1.361, *p* = .506, so the simpler model is retained. The congruency effect was significant, indicating faster overall responses to congruent relative to incongruent trials. There was also a main effect of (centered) previous RT, showing a positive relationship between previous and current RT. The main effect of PC was not significant. Critically, previous RT and congruency interacted. This negative parameter value indicates that the congruency effect got larger the faster the previous RT, as predicted by the temporal learning account. The interaction between PC and congruency remained significant, however, indicating a list-level PC effect independent of the previous RT bias. The parameter estimate for the list-level PC effect was reduced by including previous RT in the model, however. For brevity, the model without previous RT as a factor is not presented here, but the parameter for the congruency by PC interaction was .059147.

**Table 2:** Analysis 2 coefficients, standard errors, *t* values, and *p* values for congruency x proportion congruency x previous RT mixed model on inverse RTs.

Variable	Estimate	*SE*	*t*	*p*
Intercept	−1.667405	.015013	−111.067	<.001
Congruency	.234320	.007355	31.857	<.001
Proportion congruency	.008380	.023773	.395	.693
Previous RT	.185407	.008055	23.017	<.001
Congruency: Proportion congruency	.050523	.011483	4.400	<.001
Congruency: Previous RT	−.085322	.012796	−6.668	<.001

#### Previous congruency

It was further tested whether the effects of previous RT might actually be due to previous congruency. Adding previous congruency and the previous congruency by congruency interaction did not add significant variance explained to the model, *χ^2^*(2) = 4.391, *p* = .111, showing that previous congruency does not explain unique variance beyond that attributable to previous RT. Conversely, adding previous RT and the previous RT by congruency interaction to a model that includes previous congruency and previous congruency by congruency *does* result in a significant increase in variance explained, *χ^2^*(2) = 446.190, *p*<.001, thus showing that previous RT explains variance unique from previous congruency. Combined, these two results suggest that previous RTs are important in producing a list-level PC effect, whereas previous congruency is not. This follows, because if previous congruency did have a real effect on current trial congruency, then it should explain unique variance from that attributed to the only moderately correlated previous RT variable.

### Discussion

Analysis 2 demonstrated a significant interaction between previous response time and congruency. That is, the congruency effect got smaller the longer the previous response time. Importantly, this result indicates for the first time a role for temporal learning in the list-level PC effect. Inclusion of the congruency by previous RT interaction in the model reduces the parameter for the list-level PC effect. However, there was still a significant list-level PC effect independent of previous RT. It is possible, however, that temporal learning may still explain the whole PC effect, because previous RT is probably a bad estimate of temporal expectancy (as demonstrated later in the manuscript). That said, the current results are encouraging for the view that conflict adaptation might explain part of the effect, given that a significant list-level PC effect was still observed.

Analysis 2 also considered the unique hypothesis that effects of previous RT may simply be due to a confounding with previous congruency. If this were the case, it could potentially be argued that previous RT effects are not due to temporal learning, but simply to another form of conflict adaptation: a sequential congruency effect [37]. However, the model results argue against this. If the previous RT by congruency interaction was spurious, then it should have failed at explaining unique variance when adding previous congruency to the model. This was not the case. Instead, previous congruency failed to account for unique variance from that attributable to previous RT, which should not have occurred if previous congruency had any actual effect on congruency. Thus, it seems that it is the response speed of the previous trial that is important, and not congruency per se. Critically, the combined results of Analysis 2 established for the first time that the list-level PC effect is indeed confounded by temporal expectancies.

## Experiment 1

Although some readers may find the temporal learning account less intuitive than the conflict adaptation account, the current experiment will show that a (pseudo) “proportion congruent” effect can be produced even in a task with no distracters, no conflict, and no congruency manipulation. This is achieved simply by controlling the percentage of fast versus slow responses that participants make with a variable other than congruency. In particular, participants responded to a target letter that was either easily visible (high contrast) or difficult to see (low contrast). The observation of faster responses to high relative to low contrast trials is here referred to as a *contrast effect*. Note that there are fast and slow responses in this task (i.e., induced by high and low contrast, respectively), but no distracting stimuli and therefore no conflict. The proportion of high versus low contrast stimuli, termed here *proportion easy*, was then manipulated as a pseudo-PC manipulation. For half of the participants, 70% of the stimuli were high contrast (mostly easy). For the other half, 30% of the stimuli were high contrast (mostly hard). If the temporal learning account is correct, then participants will learn a faster expectancy in the mostly easy condition, resulting in a larger contrast effect, relative to the mostly hard condition, mimicking a proportion congruent effect.

Some work has already shown that mixing high and low contrast stimuli leads to a reduction of the contrast effect relative to blocked presentation of high and low contrast stimuli [25]. This shows that mixing leads to easy trials affecting hard trials, and vice versa. The most unique features of the current experiment are that (a) it is tested to what extent the difficulty (contrast) effect is affected by changes in proportions of easy trials, and, more critically (b) unlike past temporal learning work, the manipulation perfectly parallels a prototypical PC task. The only difference is that congruent and incongruent trials are replaced with high and low contrast trials, respectively. If conflict adaptation is the sole factor that produces a list-level effect, then the removal of conflict should eliminate it. Furthermore, this experiment makes it possible to explore whether such a proportion easy effect can be observed independent of the influence of the response time of the immediately preceding trial. This could therefore lend credence to the notion that the remaining list-level PC effect in Analysis 2 could still be due (in whole or in part) to temporal learning occurring across the task as a whole. In that vein, similar mixed models analyses as those presented in Analysis 2 are conducted following the main results.

### Method

#### Participants

Forty-six Ghent University undergraduates participated in Experiment 1 in exchange for €4. Participants provided written consent prior to participation. This research was approved by the ethics committee of Ghent University.

#### Apparatus

Stimulus and response timing were controlled by E-Prime software (Psychology Software Tools, Pittsburgh, PA). Responses were recorded on an AZERTY keyboard with the D, F, J, and K keys using the middle and index fingers of each hand for the stimuli “D,” “F,” “J,” and “K,” respectively.

#### Materials and design

The stimulus letters “D,” “F,” “J,” and “K” were presented on a dark grey background (RGB: 100,100,100) in uppercase, bold, 18 pt. Courier New font. On some trials the letter was presented in a high contrast whitish grey (200,200,200) and on others in a low contrast dark grey (110,110,110), thus making eight unique stimuli. Subjectively, both types of stimuli were easily visible, but more rapidly so for high contrast items. A contrast effect is the observation of slower or less accurate responses to low relative to high contrast letters. Proportion easy was manipulated between participants by having either 70% high contrast and 30% low contrast (mostly easy) or 30% high contrast and 70% low contrast (mostly hard). The experiment did not use filler and critical items, because item-specific learning is less a concern in a task with no predictive distracters, as forthcoming follow-up work will demonstrate. The experiment was run in two different locations, unintentionally with two different versions of the same experiment, but only varying in length. Ten participants saw 300 trials and the remaining saw 200 trials. No differences were observed between the groups, so the data are combined. Trials were selected at random with replacement.

#### Procedure

On each trial, participants first saw a white (255,255,255) fixation “+” for 250 ms, followed by a blank screen for 750 ms, followed by the target letter for 2000 ms or until a response was made. The next trial immediately followed correct responses. “XXX” in red (255,0,0) was presented for 500 ms following incorrect responses and trials where participants failed to respond in 2000 ms.

The mixed model analysis was identical to that in Analysis 2, including data treatments, with two small exceptions: (1) inspection of the Q-Q plots revealed no need for trimming, and (2) contrast and proportion easy replaced congruency and PC in the model. Thus, the model included contrast (high vs. low), proportion easy (mostly easy vs. mostly hard), previous RT, and their interactions as fixed factors. Subjects and items (the four letters) were again included as random factors.

### Results

Correct response latencies and percentage errors were analysed. Trials on which participants failed to respond during the 2000 ms stimulus presentation (less than 1% of the data) were deleted.

#### Response latencies

**Figure 4:**
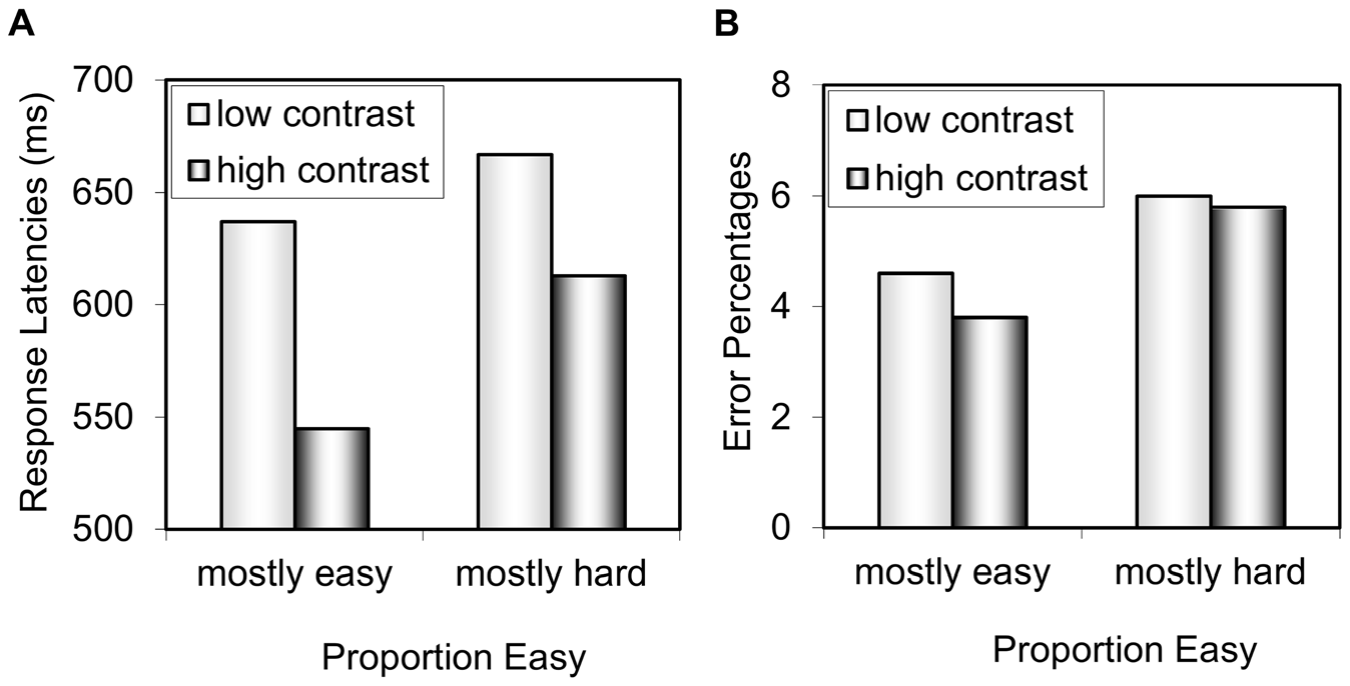
Experiment 1 data for contrast and proportion easy. Mean (A) response times and (B) error percentages.

The response latency data for Experiment 1 are presented in Figure 4. The 2 contrast (high vs. low) x 2 proportion easy (mostly easy vs. mostly hard) ANOVA revealed a significant contrast effect, *F*(1,44) = 81.514, *MSE* = 1515, *p*<.001, 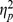 = .65, indicating faster overall responses to high contrast relative to low contrast trials. The main effect of proportion easy was not significant, *F*(1,44) = 2.942, *MSE* = 18586, *p* = .093, 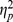 = .06. Critically, contrast and proportion easy interacted, *F*(1,44) = 5.318, *MSE* = 1515, *p* = .026, 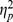 = .11, indicating a larger contrast effect in the mostly easy condition (high: 545 ms, low: 637 ms, effect: 92 ms) relative to the mostly hard condition (high: 613 ms, low: 667 ms, effect: 54 ms).

#### Percentage error

The percentage error data for Experiment 1 are also presented in Figure 4. Numerically, the errors were consistent with the response latencies, but much less sensitive. The 2 contrast (high vs. low) x 2 proportion easy (mostly easy vs. mostly hard) ANOVA did not reveal a main effect of contrast, *F*(1,44) = .871, *MSE* = 5.5, *p* = .509, 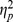 = .02, or proportion easy, *F*(1,44) = 2.222, *MSE* = 30.5, *p* = .143, 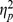 = .05. The interaction was also not significant, *F*(1,44) = .444, *MSE* = 5.5, *p* = .509, 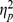
<.01.

#### Mixed models

The same linear mixed effects model as Analysis 2 was applied to the data of Experiment 1. The parameters and statistical tests are presented in Table 3. The contrast effect was significant, indicating faster overall responses to high relative to low contrast trials. There was also a main effect of previous RT, showing a positive relationship between previous and current RT. The main effect of proportion easy was significant. Critically, previous RT and contrast interacted, demonstrating that the contrast effect got smaller the slower the previous response time, again as predicted by the temporal learning view. Similar to Analysis 2, proportion easy and contrast still interacted, indicating that previous RT explains some but not all variance in the temporal learning effect. Again for brevity, the model without previous RT as a factor is not presented here, but the parameter for the stimulus contrast by proportion easy interaction was .099769. The proportion easy by contrast interaction also remained when previous contrast was considered in the model.

**Table 3:** Experiment 1 coefficients, standard errors, *t* values, and *p* values for contrast x proportion easy x previous RT mixed model on inverse RTs.

Variable	Estimate	*SE*	*t*	*p*
Intercept	−1.825381	.044080	−41.410	<.001
Contrast	.165333	.013208	12.517	<.001
Proportion easy	−.128134	.061775	−2.074	.043
Previous RT	.123592	.013313	9.284	<.001
Contrast: Proportion easy	.092703	.018698	4.958	<.001
Contrast: Previous RT	−.041207	.017721	−2.325	.020

### Discussion

This experiment provided suggestive evidence for temporal learning in the context of a PC-like task manipulation. In the context of a task where most of the trials take quite awhile to respond to (mostly hard), participants are more prepared to respond slowly to the frequent low contrast targets. This impairs a participant's ability to produce an (unexpected) fast response to high contrast trials, thus resulting in a small contrast effect. However, when most of the trials in the task can be responded to quickly (mostly easy), participants are more prepared to respond quickly to the frequent high contrast targets. This leads to fast responses to these easily-identifiable targets, thus resulting in a larger contrast effect. Note that with no main effects of proportion easy, this “proportion easy” effect is not driven by scaling (i.e., larger effects with increasing response times and errors). Evidence for this sort of temporal learning has been observed before [3], [28]. Most critically for the topic of the current report, however, this experiment has the novel feature of having an identical task structure as a PC experiment, save that congruent trials are replaced with high contrast trials and incongruent trials with low contrast trials. Of course, this contrast experiment is only analogical to the list-level PC paradigm and cannot be used to draw strong conclusions about the list-level PC effect. Along with the mixed model analyses of Analysis 2, this experiment does shows how temporal learning might contribute to the list-level PC effect independent of any conflict.

The mixed modelling added two other important contributions. First, the results confirmed a role of the immediately preceding trial in developing the proportion easy effect. Importantly, a significant proportion easy effect still remained after controlling for response times on the preceding trial. As hinted at in the previous analysis, this is probably because previous RT is only a very noisy estimate of temporal expectancy. This is an interesting finding in the current task context, because the remaining proportion easy effect observed here presumably cannot be interpreted as conflict adaptation, an inference one might like to draw from the data of Analysis 2 where the same weak effect of previous RT was observed. Again, no strong conclusions about the list-level PC effect can be drawn from this contrast experiment, but the results do hint that the role of temporal learning may be much larger than what the previous RT variable suggests.

## Analysis 3: Simulated Previous RT

Analysis 2 demonstrated that temporal learning confounds do contribute to the list-level PC effect. To reiterate, this was indexed by larger congruency effects with faster previous RTs. However, while including previous RT in the regression did reduce the size of the list-level PC effect, it only did so by a small amount. The significant remaining list-level PC effect might seem to indicate that list-level conflict adaptation exists on top of any observed temporal learning. This could be the case. On the other hand, the same thing was observed with the proportion easy effect of Experiment 1: previous RT explained some of the effect, but not all of it.

Why could this be? Previous RT is used as a proxy for temporal learning. Although previous RT should correlate somewhat with a participant's temporal expectancy, how strong will this correlation be? To assert that previous RT is a perfect measure of temporal expectancy requires the assumption that the relation between previous RT and the congruency effect is linear (i.e., due to the way regression works). In other words, it assumes that for every *x* decrease in previous RT, there should be a *y* increase in the size of the congruency effect. This is almost certainly not the case. Furthermore, temporal expectancies are likely based on more than just the previous trial, meaning that previous RT will only be loosely correlated with a participant's actual temporal expectancy (e.g., consider that a given previous RT corresponds to *one* of the tick marks in Figure 1, and potentially one of the outlying ones). Noisiness in temporal expectancies will further reduce the explanatory power of previous RT. If previous RT is only *partially* correlated with temporal expectancy, then the previous RT variable will only explain *part* of the variance due to temporal learning. What it misses, the PC by congruency (or proportion easy by contrast) interaction will continue to soak up. This latter result is a problem of multicollinearity.

Indeed, the goal of the current analysis is to assess whether previous RT eliminates the list-level PC effect in the simulated data created with the PEP model in Analysis 1. This is an interesting question, because it is known a priori that the PEP model produces the PC effect via temporal learning. If a list-level PC is still observed after controlling for previous RT, this will demonstrate why conflict adaptation is not the only possible interpretation.

### Method

The simulated data from Analysis 1 were subjected to the same mixed modelling procedure as Analysis 2, with two exceptions: (1) items were a priori identical in the model and were therefore not entered as a random factor, and (2) inspection of the Q-Q plots of the inverse cycle times (i.e., simulated RTs) revealed no need for trimming.

### Results

The results for the full model are presented in Table 4. Note again that the parameter estimates are based on inverse response times, which are not easily transformed back into normal response times. As can be seen, the model produced a significant congruency effect. The main effect of PC was also significant. The main effect of previous RT was significant, indicating that current and previous RTs were positively correlated. Importantly, previous RT and congruency interacted. The negative sign of this parameter means that the congruency effect got larger the faster the previous response time, consistent with the temporal learning view. Critically, PC and congruency interacted, indicating a PC effect even after factoring out the influence of previous RT on congruency. Furthermore, removing PC as a factor from the regression (i.e., along with its interaction with congruency) significantly decreased the amount of variance explained, *χ^2^*(2) = 51.504, *p*<.001. This means that the model finds evidence for a PC effect that goes beyond what previous RT can explain. There was also a significant loss in variance when removing previous RT as a factor from the full model, *χ^2^*(2) = 54.692, *p*<.001. This indicates that previous RT does at least capture some of the temporal learning effect. However, the parameter for the PC effect for this latter model (.035452) was only reduced somewhat in the full model (.032729), similar to the real data, which is not particularly impressive. In other words, previous RT misses a *majority* of the variance in the PC effect that temporal learning is known to produce in the PEP model.

**Table 4:** Analysis 3 coefficients, standard errors, *t* values, and *p* values for a congruency x proportion congruency x previous RT mixed model on inverse RTs.

Variable	Estimate	*SE*	*t*	*p*
Intercept	−2.828764	.003058	−925.129	<.001
Congruency	.687471	.004341	158.354	<.001
Proportion congruency	−.031107	.004348	−7.168	<.001
Previous RT	.018307	.002680	6.831	.005
Congruency: Proportion congruency	.032729	.006174	5.301	<.001
Congruency: Previous RT	−.010639	.003808	−2.794	.005

### Discussion

The results of Analysis 3 are clear. Firstly, the results confirm that previous RT can be used to detect whether or not temporal learning is playing a role in the data. However, the results also demonstrate that previous RT misses the majority of the variance attributable to temporal learning. That is, it is known a priori that the PEP model produces the entire list-level PC effect via temporal learning processes, but using previous RT as a control measure of temporal learning does not eliminate the PC effect. When observing this in real participants (e.g., Analysis 2) there might be a temptation by the experimenter to interpret this remaining PC effect as evidence for list-level conflict adaptation. In the simulated data, however, this is known to be an impossible conclusion: the model has no conflict adaptation device. Previous RT is simply a very poor measure of temporal learning that will only explain a small fraction of the variance actually due to temporal learning processes. Of course, these observations do not exclude the possibility that conflict adaptation also occurs (it very well may), but they tell a cautionary tale about interpretations of list-level PC effects, even when measures have been taken to factor out the influence of previous response times.

## General Discussion

The results of the three analyses and one experiment presented here are both clear and ambiguous. They are clear in demonstrating that a temporal learning bias is present in list-level PC, but ambiguous as to how large of a bias there is. Conflict adaptation may very well still play a role. Ambiguity aside, if the list-level PC effect is to be taken as evidence of conflict adaptation, then such an effect should not be confounded with other things, such as temporal expectancy. The current manuscript utilized three approaches to make the case that concern over temporal confounds is warranted. First, Analysis 1 presented a modified version of the PEP model to demonstrate that temporal learning could, in theory, produce a list-level PC effect.

Second, Analysis 2 showed that the length of response times on previous trials was negatively related to the congruency effect with actual participants, consistent with related findings from the Kinoshita lab [28]. That is, with increasing previous response times the congruency effect got smaller. Critically, this was observed for the first time with contingency-unbiased list-level PC, and also for the first time with supraliminal, integrated stimuli. Including previous RT in the linear mixed effects model reduces, but does not eliminate, the list-level PC effect. However, it is again worth pointing out that the previous RT variable is probably a very weak proxy of temporal expectancy, as the Analysis 3 results on the modelled data illustrate.

Third, Experiment 1 used a contrast (rather than congruency) manipulation to show that learning about how fast to respond in a task can account for larger effects in a mostly easy task relative to a mostly hard task, even when there is no conflict to adapt to. The novel feature of this particular design is that it parallels a prototypical PC experiment, but removes conflict from the design. Of course, the observed “proportion easy” effect does not rule out conflict adaptation as an additional mechanism in the PC task. Rather, the proportion easy effect demonstrates the more general point that between-participant manipulations that allow for expectancies of when to respond can have profound effects on the results. This has already been demonstrated repeatedly in the temporal learning literature [6], [19], but is a critical consideration for assessing list-level PC effects. Differences in difficulty do not always produce this sort of interaction, however [3], which might indicate that temporal learning only influences behaviour in certain contexts. In future work, if a context can be determined in which temporal learning is not engaged during a list-level PC task, then this might serve to resolve some of the ambiguities raised in the current report.

Overall, the combined results suggest a presence of temporal learning in this sort of task. Whether *all* of the list-level PC effect is explained by this temporal learning is ambiguous, however, because a complete dissociation of proportion congruency and temporal learning was not possible in this work. This is a tricky issue to disentangle, given how inherently confounded PC and the average speed of responses are. Indeed, this is a general problem for the conflict adaptation literature [13]. Task regularities have to be manipulated to create variables such as proportion congruency, which provide various sources of information (many unintended by the researcher) for the participant to learn. Although the one-process temporal learning account is more parsimonious, there could nevertheless be other active processes (e.g., conflict adaptation) playing a role. The critical implication of the current work, however, is that the list-level PC effect is confounded with temporal expectancies and this muddies the interpretation of which process(es) explain the effect.

One possible mechanistic explanation for temporal learning, discussed in the Introduction and modelled in Analysis 1, is basically identical to the account Schmidt has previously given for contingency learning [38] and evaluative conditioning [39], with the addition of a role for temporal information. Other temporal learning accounts, such as the ASE model, can equally well explain the list-level PC effect with mechanisms that are also unrelated to conflict adaptation. The current results therefore do not argue for or against any *specific* version of the temporal learning account, but instead argue that temporal learning of some sort plays a role. One benefit of the account suggested here, however, is that both contingency and temporal learning are explainable by the same memory storage and retrieval processes. Additionally, there may be more to temporal learning than anticipating accuracy (i.e., as in the ASE model). To use the example of music again, one does not aim to play the series of notes in a song as quickly and accurately as possible, but to play each note at the correct time. Future research on these nuanced issues could prove informative.

### Limitations

Of course, a notable limitation of the contrast experiment is that one must make a cross-paradigm inference to draw any firm conclusions. One might argue, for instance, that the proportion easy interaction produced in Experiment 1 is driven by something entirely different than list-level PC effects. For instance, participants might squint more in the mostly hard task to better perceive the frequent low contrast items, and this could be what results in a reduced contrast effect. It is also notable that the pattern of the interaction appears a bit different, with the proportion easy effect of Experiment 1 seemingly driven by changes in the high contrast condition (though this was not true in subsequent experiments in our lab not presented here), whereas the PC effect of Hutchison [18] is seemingly driven by changes in the incongruent condition (though this does not always seem to be the case, either [9]). These inconsistencies might indicate that something different, such as conflict adaptation, occurs on top of the temporal learning effect with list-level PC. One might additionally argue that low contrast stimuli create relatively more perceptual conflict, which perhaps does not rule out a conflict adaptation account entirely for such results. Although less parsimonious, two different mechanism (i.e., temporal learning and conflict adaptation) may still be required to explain all of the data.

It is worth noting that Hutchison also observed an effect of working memory capacity (WMC) on the list-level PC effect, with a larger effect for low relative to high WMC participants [18]. This was argued as evidence that high WMC participants are generally good at staying on task all of the time, whereas low WMC participants are more likely to allow attention to stray to the distracting word in the mostly congruent condition. Such WMC effects, while beyond the scope of the current work, are equally well explainable in terms of temporal learning. It has been demonstrated, for instance, that high WMC participants are generally quite good at focusing on the target task, whereas low WMC participants are more likely to attend to and learn about task-irrelevant information such as time [40]. Thus, temporal learning effects should be larger for low WMC participants. Indeed, note that both accounts of WMC effects are essential identical, save for the proposed distracting information that inattentive low WMC participants are being influenced by (i.e., conflict vs. temporal information). This might also explain the finding of Hutchison that item-specific PC effects were larger for low WMC participants (i.e., low WMC participants attend more to the task-irrelevant contingencies). Future work on these issues could therefore prove informative.

Hutchison also observed, however, a larger *item-specific* PC effect for participants in the (list-level) mostly congruent condition [18]. It is less clear how a temporal learning mechanism might produce this result. It could be that there is some sort of interaction between contingency and temporal learning, whereby contingencies have a larger effect when in a mostly easy context. However, a post hoc ANOVA on the Analysis 1 simulated data revealed an overall item-specific PC effect, *F*(1,1998) = 5.432, *MSE* = 1919.561, *p* = .020 (incidentally, this demonstrates backward compatibility with the original modelling results), but no interaction with list-level PC, *F*(1,1998) = .494, *MSE* = 1957.154, *p* = .482. Thus, at least without changes to the model, the PEP does not replicate this specific finding of Hutchison. Consequently, the modulation of item-specific PC by list-level PC observed by Hutchison seems to indicate clearer evidence for conflict adaptation (though, of course, not of a completely list-level nature).

It is important to highlight the fact that stimulus contrast was not in any way predictive of *what* response to make in Experiment 1. One of the two types of stimulus contrast were more likely depending on which condition the participant was in, but stimulus contrast does not tell a participant anything about whether the D, F, J, or K keys should be pressed. If anything, stimulus contrast (or luminance) could have served as a contextual cue allowing participants to adjust their temporal expectation about when to respond on a trial-by-trial basis. Context-level learning is frequently observed in the cognitive control literature [41]–[43], and any sort of context-level temporal learning would have actually blurred the difference between the mostly easy and mostly hard conditions, thus artificially *reducing* the proportion easy effect. The same problem is unlikely in the proportion congruent task, because congruency probably cannot serve as a contextual cue in this same way. A related question that future work could aim to answer is whether context-level temporal learning can indeed occur in this sort of paradigm.

### Statistical Caveat

One limitation of the mixed models approach used in this paper is that temporal expectancy was operationalized as previous RT, which Analysis 3 and other research [6] suggests is probably a very poor measure. Previous RT is likely to miss large quantities of variance that it should account for. Thus, temporal expectancy is measured in an overly conservative way. Because of this problem, the list-level PC interaction term can steal some of this missed variance, a statistical principle demonstrated clearly in Analysis 3. Indeed, because list-level PC is almost by definition strongly correlated with previous reaction times (i.e., because more trials are congruent/fast in the mostly congruent block relative to the mostly incongruent block) the regressor for the list-level PC effect will be extremely effective at capitalizing on this variance. In other words, in addition to any (potential) conflict adaptation biases, the binary list-level PC regressor can accumulate the temporal learning biases that occur across trials that variables like previous RT fail to capture. Thus, the measure of conflict adaptation is extremely liberal, making it difficult to interpret the remaining list-level PC effect found in the current work.

Note that the reverse problem is not true. Variables like previous RT can explain temporal expectancy biases that occur consistently across the mostly congruent and mostly incongruent conditions, but cannot explain systematic differences occurring between the two PC conditions. Thus, the conflict adaptation measure can be confounded by temporal learning biases, but not vice versa. As an aside, it could also in principle happen that previous congruency would steal variance from previous RT in the same way and for the same reasons. The comparisons in the “Previous Congruency” section of Analysis 2 revealed no statistically-significant evidence for this, but if observed in future research it should be interpreted with caution for the reasons outlined above. In statistical terms, these issues are problems of multicollinearity.

### Conclusions

The present work explored an alternative interpretation of the list-level proportion congruent effect. With a combination of computational modelling, statistical modelling, and experimental results, it was demonstrated that learning when to respond contributes to the list-level PC effect. Unfortunately, it is difficult to know at present whether temporal learning is the whole story, especially in light of the Analysis 3 modelling results. Indeed, none of the current results directly argue against the possibility of list-level conflict adaptation. Future research is therefore needed to find more refined ways of dissociating temporal expectancy and conflict adaptation biases. Nevertheless, the critical contribution of the current work is the cautionary demonstration that the list-level PC effect cannot be taken as strong evidence for conflict adaptation without further controls.

## Supporting Information

Appendix S1 **Major and minor model changes.**
(DOCX)

## References

[1] Schmidt JR (2012) Human contingency learning. In: Seal NM, editor. Encyclopedia of the sciences of learning. New York: Springer. pp. 1455–1456.

[2] Matzel LD, Held FP, Miller RR (1988) Information and expression of simultaneous and backward associations: Implications for contiguity theory. Learning Motiv 19: 317–344.

[3] Los SA (1999) Identifying stimuli of different perceptual categories in mixed blocks of trials: Evidence for cost in switching between computational processes. J Exp Psychol Human 25: 3–23. 10.1037//0096-1523.25.1.3 10069025

[4] Los SA (1999) Identifying stimuli of different perceptual categories in pure and mixed blocks of trials: evidence for stimulus-driven switch costs. Acta Psychol 103: 173–205. 10.1016/s0001-6918(99)00031-1 10555490

[5] Taatgen N, Van Rijn H (2011) Traces of times past: Representations of temporal intervals in memory. Mem Cogn 39: 1546–1560. 10.3758/s13421-011-0113-0 PMC3205264 21626068

[6] Van Maanen L, Brown SD, Eichele T, Wagenmakers EJ, Ho T, et al (2011) Neural correlates of trial-to-trial fluctuations in response caution. J Neurosci 31: 17488–17495. 22131410 10.1523/JNEUROSCI.2924-11.2011 PMC6623798

[7] Stroop J (1935) Studies on interference in serial verbal reactions. J Exp Psychol 18: 643–662.

[8] Botvinick MM, Braver TS, Barch DM, Carter CS, Cohen JD (2001) Conflict monitoring and cognitive control. Psychol Rev 108: 624–652. 11488380 10.1037/0033-295x.108.3.624

[9] Cheesman J, Merikle PM (1986) Distinguishing conscious from unconscious perceptual processes. Can J Psychol 40: 343–367. 3502878 10.1037/h0080103

[10] Lindsay DS, Jacoby LL (1994) Stroop process dissociations: The relationship between facilitation and interference. J Exp Psychol Human 20: 219–234. 10.1037//0096-1523.20.2.219 8189189

[11] Lowe DG, Mitterer JO (1982) Selective and divided attention in a Stroop task. Can J Psychol 36: 684–700. 7159848 10.1037/h0080661

[12] Jacoby LL, Lindsay DS, Hessels S (2003) Item-specific control of automatic processes: Stroop process dissociations. Psychon B Rev 10: 638–644. 10.3758/bf03196526 14620358

[13] Schmidt JR (2013) Questioning conflict adaptation: Proportion congruent and Gratton effects reconsidered. Psychon B Rev 20: 615–630. 10.3758/s13423-012-0373-0 23325703

[14] Schmidt JR (2013) The Parallel Episodic Processing (PEP) model: Dissociating contingency and conflict adaptation in the item-specific proportion congruent paradigm. Acta Psychol 142: 119–126. 10.1016/j.actpsy.2012.11.004 23261421

[15] Schmidt JR, Besner D (2008) The Stroop effect: Why proportion congruent has nothing to do with congruency and everything to do with contingency. J Exp Psychol Learn 34: 514–523. 10.1037/0278-7393.34.3.514 18444752

[16] Schmidt JR, Crump MJC, Cheesman J, Besner D (2007) Contingency learning without awareness: Evidence for implicit control. Conscious Cogn 16: 421–435. 16899377 10.1016/j.concog.2006.06.010

[17] Bugg JM, McDaniel MA, Scullin MK, Braver TS (2011) Revealing list-level control in the Stroop task by uncovering its benefits and a cost. J Exp Psychol Human 37: 1595–1606. 10.1037/a0024670 PMC3609544 21767049

[18] Hutchison KA (2011) The interactive effects of listwide control, item-based control, and working memory capacity on Stroop performance. J Exp Psychol Learn 37: 851–860. 10.1037/a0023437 21517220

[19] Los SA (1996) On the origin of mixing costs: Exploring information processing in pure and mixed blocks of trials. Acta Psychol 94: 145–188.

[20] Grice GR (1968) Stimulus intensity and response evocation. Psychol Rev 75: 359–373. 4879423 10.1037/h0026287

[21] Kohfeld DL (1968) Stimulus intensity and adaptation level as determinants of simple reaction time. J Exp Psychol 76: 468–473. 5642161 10.1037/h0021285

[22] Ollman RT, Billington MJ (1972) The deadline model for simple reaction times. Cognitive Psychol 3: 311–336.

[23] Strayer DL, Kramer AF (1994) Strategies and Automaticity: 1. Basic findings and conceptual-framework. J Exp Psychol Learn 20: 318–341.

[24] Strayer DL, Kramer AF (1994) Strategies and automaticity: 2. Dynamic aspects of strategy adjustment. J Exp Psychol Learn 20: 342–365.

[25] Van Duren LL, Sanders AF (1988) On the robustness of the additive factors stage structure in blocked and mixed choice reaction designs. Acta Psychol 69: 83–94. 10.1016/0001-6918(88)90031-5 3245478

[26] Penney TB, Allan LG, Meck WH, Gibbon J (1998) Memory mixing in duration bisection. In: Rosenbaum DA, editor. Timing of behavior: Neural, psychological, and computational perspectives. Cambridge, MA: MIT Press. pp. 165–193.

[27] Grosjean M, Rosenbaum DA, Elsinger C (2001) Timing and reaction time. J Exp Psychol Gen 130: 256–272. 11409103 10.1037//0096-3445.130.2.256

[28] Kinoshita S, Mozer MC, Forster KI (2011) Dynamic adaptation to history of trial difficulty explains the effect of congruency proportion on masked priming. J Exp Psychol Gen 140: 622–636. 21707205 10.1037/a0024230

[29] Jones M, Mozer MC, Kinoshita S (2009) Optimal response initiation: Why recent experience matters. In: Koller D, Schuurmans D, Bengio Y, Bottou L, editors. Advances in neural information processing systems 21. Cambridge, MA: MIT Press. pp. 785–792.

[30] Mozer MC, Kinoshita S, Davis C (2004) Control of response initiation: Mechanisms of adaptation to recent experience. Proceedings of the twenty sixth conference of the Cognitive Science Society. Hillsdale, NJ: Erlbaum Associates. pp. 981–986.

[31] Mozer M, Colagrosso M, Huber D (2002) A rational analysis of cognitive control in a speeded discrimination task. In: Dietterich T, Becker S, Ghahramani Z, editors. Advances in neural information processing systems 14. Cambridge, MA: MIT Press. pp. 51–57.

[32] Kinoshita S, Lupker SJ (2003) Priming and attentional control of lexical and sublexical pathways in naming: A reevaluation. J Exp Psychol Learn 29: 405–415. 10.1037/0278-7393.29.3.405 12776751

[33] Kinoshita S, Forster KI, Mozer MC (2008) Unconscious cognition isn't that smart: Modulation of masked repetition priming effect in the word naming task. Cognition 107: 623–649. 18206138 10.1016/j.cognition.2007.11.011

[34] Mayr U, Awh E, Laurey P (2003) Conflict adaptation effects in the absence of executive control. Nat Neurosci 6: 450–452. 12704394 10.1038/nn1051

[35] Mordkoff JT (2012) Observation: Three reasons to avoid having half of the trials be congruent in a four-alternative forced-choice experiment on sequential modulation. Psychon B Rev 19: 750–757. 10.3758/s13423-012-0257-3 22549895

[36] Schmidt JR, De Houwer J (2011) Now you see it, now you don't: Controlling for contingencies and stimulus repetitions eliminates the Gratton effect. Acta Psychol 138: 176–186. 10.1016/j.actpsy.2011.06.002 21745649

[37] Gratton G, Coles MGH, Donchin E (1992) Optimizing the use of information: Strategic control of activation of responses. J Exp Psychol Gen 121: 480–506. 1431740 10.1037//0096-3445.121.4.480

[38] Schmidt JR, De Houwer J, Besner D (2010) Contingency learning and unlearning in the blink of an eye: A resource dependent process. Conscious Cogn 19: 235–250. 20116294 10.1016/j.concog.2009.12.016

[39] Schmidt JR, De Houwer J (2012) Contingency learning with evaluative stimuli: Testing the generality of contingency learning in a performance paradigm. Exp Psychol 59: 175–182. 22411181 10.1027/1618-3169/a000141

[40] Woehrle JL, Magliano JP (2012) Time flies faster if a person has a high working-memory capacity. Acta Psychol 139: 314–319. 10.1016/j.actpsy.2011.12.006 22246199

[41] Bugg JM, Jacoby LL, Toth JP (2008) Multiple levels of control in the Stroop task. Mem Cognition 36: 1484–1494. 10.3758/MC.36.8.1484 PMC2682765 19015507

[42] Crump MJC, Gong ZY, Milliken B (2006) The context-specific proportion congruent Stroop effect: Location as a contextual cue. Psychon B Rev 13: 316–321. 10.3758/bf03193850 16893001

[43] Crump MJC, Milliken B (2009) The flexibility of context-specific control: Evidence for context-driven generalization of item-specific control settings. Q J Exp Psychol 62: 1523–1532. 10.1080/17470210902752096 19370487

