# Supplementary material for: Temporal Learning and List-Level Proportion Congruency: Conflict Adaptation or Learning When to Respond?
Source: PLoS One. 2013 Nov 28;8(11):e82320. doi: 10.1371/journal.pone.0082320 (PMC3842973; doi:10.1371/journal.pone.0082320)
Supplement: Appendix S1 — Major and minor model changes. (DOCX) [file pone.0082320.s001.docx]

**Appendix S1**

**Key model changes.** At a more abstract level, the response threshold in the model drops as the cycle time (i.e., simulated millisecond of the trial) approaches the stored response times of previously encountered trials. In addition, the influence of a given episode is decreased the longer ago it occurred. More concretely, the response threshold on a given processing cycle was determined with the formula,

$threshold=baseline-\left(\sum_{i=1}^{n} \left(proximity_{i}\times strength_{i}\right)-.01\right)$ (1)

The *threshold* is restrained between the *baseline* threshold of .45 and a minimum drop to .30. Note that the threshold only drops after the summed (*proximity* x *strength*) scores exceed .01. The proximity of an episode *i* is determined with the formula,

$proximity_{i}=1-\left(\frac{\left(cycle-rt_{i}\right)^{2}}{10000}\right)$ (2)

The given *proximity* value of each episode is restricted between 0 and 1. This formula works such that when the current cycle time (*cycle*) is very close to the stored response time of the episode (*rt*), there is a large effect on the response threshold. The further apart they are, the more rapidly the influence of the node drops off (and no longer has an influence with a difference of 100 or more cycles). The strength of episode *i* is determined by the formula,

$strength_{i}=\frac{\left(40-lag_{i}\right)^{2}}{50000}$ (3)

In this formula, *lag* represents how many trials previously a given node *i* occurred. These formulas are only applied to the most recent 40 trials, so *strength* can vary from .03042 (i.e., the most recent episode) down to 0 (i.e., episodes from 40 trials back or more). Note that the influence of a node is decreased logarithmically the further ago it occurred, such that the most recent episode has an especially large impact, and this influence decreases at a decelerating rate for increasingly longer lags.

**Minor model changes.** A few other minor adjustments to the model were made. Because the new temporal learning mechanism serves to decrease the response threshold on essentially all trials (i.e., at least a little bit), the baseline response threshold was increased from .40 to .45. The maximum amount of episodic retrieval allowed was determined to be far too liberal (which led to far too fast responses to the 100% contingency filler items), and was thus decreased from .20 to .01.

An odd bimodal pattern in the response time distribution was discovered. Though the key findings of the following simulation were also produced without fixing this, it was opted to make some changes to the lower end of the model to repair it. In the old model, the *bias* (i.e., input noise) scores selected for each Input node were selected from a flat random distribution. In the new version, they were selected from a normal distribution (created by averaging three uncorrelated random variables) with the same range. This resulted in fewer outlying starting *bias* scores, which reduced the error rate. Output from word Input nodes to Identity nodes was doubled to compensate. Otherwise, the model was identical to that used by Schmidt [[14](#_ENREF_12)]. Again, these minor changes were not required to produce the predicted effects, but do produce a more realistic response time distribution. In general, it should be noted that the model results are robust to reasonable alterations in the parameters. Parameters were only adjusted to ensure that model functions worked as intended and that the model produced roughly familiar response time and error rates.
